# Survey of the Situation of Infertile Women Seeking In Vitro Fertilization Treatment in China

**DOI:** 10.1155/2013/179098

**Published:** 2013-12-03

**Authors:** Xuan Jin, Gongxian Wang, Sisun Liu, Jing Zhang, Fang Zeng, Yun Qiu, Xiaojin Huang

**Affiliations:** ^1^Center for Assisted Reproduction, The First Affiliated Hospital of Nanchang University, Nanchang, Jiangxi 330006, China; ^2^Department of Urology, The First Affiliated Hospital of Nanchang University, Nanchang, Jiangxi 330006, China; ^3^Department of Obstetrics and Gynaecology, The First Affiliated Hospital of Nanchang University, Nanchang, Jiangxi 330006, China

## Abstract

*Background*. In previous studies, people's knowledge of reproductive health and infertile women's psychological states was surveyed in several countries. However, there has been limited information concerning the psychological states of infertile women seeking treatment and the outcomes of in vitro fertilization (IVF) in China. *Methods*. Infertile women were asked to complete short questionnaires on the day that their oocytes were retrieved; these questionnaires covered the durations of their infertility, levels of education, sources of pressure, and psychological states. Data concerning IVF outcomes were provided by embryologists and clinicians. The correlations between the duration of infertility and educational level, psychological state and education level, and psychological state and outcome of IVF were analyzed in the cohort study. *Results*. The duration of infertility in more than half of the females was longer than 5 years. Compared with less-educated women, women with higher levels of education sought treatment earlier and their rates of depressive symptoms were lower. There is an association between negative emotions and outcome of IVF. *Conclusions*. The survey of the situations of infertile women seeking IVF treatment in China indicates the importance of popularizing knowledge concerning reproductive health. Improving medical conditions, reducing the costs of treatment, and developing social culture will aid in relieving the stress of infertile women and improving assisted reproductive treatment.

## 1. Introduction

Childbirth is considered a major component of human life; most men and women take parenthood for granted and look forward to it. A couple is generally considered clinically infertile when pregnancy has not occurred after at least twelve months of regular unprotected sexual activity [1]. Infertility affects 72.4 million people worldwide and has been named a major medical and social problem by the World Health Organization [2].

The inability to conceive children is highly stressful for individuals and couples, and women bear the brunt of the reaction from society in most cases. In Mozambique, certain social activities and traditional ceremonies are forbidden for infertile women [3], while in Brazil, in a “life without children,” the quality of the marital relationship would be considered irregular [4]. In India it is reported that 70% of women experiencing infertility are punished with physical violence for their “failure” [5]. In China, especially in the countryside, where social health insurance is less than ideal, children, especially sons, are regarded as sources of income and security in old age. Women who are unable to give birth tend to be involved in marital conflict, and a part of them face the threat of divorce [6].

Anxiety and depression are common emotional responses among couples with infertility. In a Chinese prevalence study, 23.2% of infertile women met the criteria for generalized anxiety disorder and 17% for major depressive disorder [7]. Negative emotions in infertile patients are much more common than those in fertile women [8]. To create the possibility of biological parenthood for couples who would otherwise have remained childless, various effective infertility treatments and assisted reproductive technologies (ART) have presently been established. For most couples experiencing infertility, in vitro fertilization (IVF) or intracytoplasmic sperm injection (ICSI) is a last resort, although a small number of infertile couples drop out and are denied further fertility treatment in an IVF/ICSI program for various reasons. According to a 2006 report by the International Committee Monitoring Assisted Reproductive Technology, more than 3 million babies worldwide have been conceived through either IVF or ICSI [9]. A relationship between psychological factors and IVF/ICSI has been described in many previous studies, but the results were not highly consistent.

In addition to psychological factors, many biomedical factors, including the age of the woman, the duration of infertility, previous IVF treatment, the number of oocytes retrieved, and the quality of embryos transferred, are important predictors of IVF outcome [10].

Knowledge of infertility and its treatment can be determining factors to a certain degree. However, knowledge is inadequate in many places around the world according to a global survey of 17,500 women of childbearing age from 10 countries [11]. Research has been conducted exploring the knowledge, behaviors, and practices surrounding infertility. For example, the mean duration of infertility in women seeking IVF is different in various countries and places. In India, the data suggests a mean of approximately 8.38 years [12]; in Korea, it is approximately 4 years [13]; in Italy, more than half of women would seek ART following periods of infertility lasting less than 3 years [14].

Exploration of the societal perceptions of infertility can assist clinical work, providing better help to infertile couples. However, in China, only limited data are available. The purpose of this study is to achieve a better understanding of the infertile Chinese population's awareness of infertility and certain treatment options (i.e., assisted reproductive technologies). To make this research more clear, the relationship between psychological stress and IVF outcome will also be described.

## 2. Methods

### 2.1. Ethics and Recruitment

In China, IVF treatment can only be provided by licensed centers. This study was approved by the Human Research Ethics Committee at the First Affiliated Hospital of Nanchang University. Participants were recruited from the IVF center at the First Affiliated Hospital of Nanchang University. Women with an indication for IVF or ICSI treatment according to the Ministry of Health Monitoring Human Assisted Reproductive Technology Management [15], and in the process of starting the first treatment were eligible to participate in the study.

From February 2012 to August 2012, a total of 460 women receiving IVF/ICSI treatments in the center were all invited to enroll in the study; the participants aged from 22 to 41 years.

### 2.2. Psychological Measures

In this study, to investigate the participant's basic sociodemographic characteristics, psychological data, and fertility history, a short questionnaire that included both structured and open-ended questions was completed by each participant on the day their oocytes were retrieved; such data as location, age, educational level, employment, duration of infertility, medical indications for treatment, and major source of pressure (family versus society; the attitudes of family members including those of elderly relatives and/or husbands, while societal pressure included discrimination) were gathered. Depressive and anxious symptoms were evaluated using the Zung Self-Rating Depression Scale (ZDS) and the Zung Self-Rating Anxiety Scale (ZAS) [16, 17]. There are 20 positively and negatively formulated items, which cover affective, psychological, and somatic symptoms of depression and anxiety. The answer to each item is coded on a four-point scale to indicate the frequency of the symptoms. Negative items are converted into positive ones with total scores ranging from 20 to 80; scores of <40 indicate nondepressive or nonanxious symptoms, while scores ≥40 indicated depressive or anxious symptoms [18]. The items addressing helplessness regarding fertility problems were obtained from the Illness Cognition Questionnaire (ICQ) for IVF patients [19]; the cut-off score of 14 and above indicated helplessness [20]. The psychological dataset was matched with the IVF/ICSI dataset.

### 2.3. IVF/ICSI Treatment

Ovarian stimulation is routinely induced using a long protocol, starting with gonadotropin-releasing hormone agonist (GnRH-a, Decapeptyl, Ferring AG) for pituitary downregulation followed by gonadotropin- (Gn-) like recombinant follicle stimulating hormone (FSH, Gonal-F, Serono) for follicles stimulation; when two or more follicles greater than 18 mm in diameter are associated with a consistent rise in serum estradiol, urinary human chorionic gonadotropin (hCG, Serono) is used. Oocytes retrieval is subsequently performed 35 to 37 hours after hCG is administered. Fertilization is assessed by a two-pronuclear stage 16 to 18 hours after insemination; excellent embryos are defined as those with blastomeres of more than 6 cells with percentages of fragmentation less than 10% and varying in size by a factor of blastomere regularity that is less than 2 at day 3, as determined by the embryologists' standard clinical practice [21]. To reduce multiple clinical pregnancies, two embryos were transferred when the woman's age is less than 35 years, and three embryos were transferred when the age of woman is more than 35 years. Clinical pregnancy is defined as an ultrasound-verified pregnancy 5 weeks after the embryos have been transferred.

### 2.4. Statistical Analysis

The data were analysed with SPSS software (version 11.5). Associations were assessed using a chi-square test and correlation and regression analyses, and the mean number of oocytes was compared using a *t*-test. *P* values of <0.05 were considered to be statistically significant.

## 3. Results

### 3.1. Realization of the Need for IVF/ICSI Treatment

A total of 460 women from infertile couples receiving IVF or ICSI treatment participated in this survey; more than half of the women began IVF treatment when their infertility had lasted longer than 5 years. Only approximately 10% would seek IVF treatment when the duration of their infertility was less than 2 years (Table 1).

Table 2 shows the relationships between educational level and the duration of infertility in women seeking IVF/ICSI treatment for the first time. In the comparative analysis, when the duration of infertility was less than or equal to one year, there was no difference between the women with different educational levels, and in the group with durations between 1 and 2 years; the women who had completed high school were outnumbered by those with primary school and university/college education levels (6.9% versus 9.5% and 10.2%, resp.). The total number of women seeking treatment following periods of infertility of less than 2 years was 52, composing approximately 11.3% of all 460 participants. We found that half of the women with university or college education levels chose IVF treatment after having suffered from 2 to 5 years of infertility in contrast to only 14.3% of the women with primary school education levels and 33% with high school education levels (*P* < 0.01). However, there was not a statistically significant difference between the women with primary and high school education levels. Among the 63 participants with primary school education, about three-quarters of them did not seek the help of ART until they had experienced more than 5 years of infertility; the longest duration of infertility reported was 18 years (data not shown). The number of women with infertility lasting more than 5 years significantly decreased with increasing educational levels (primary school versus high school, *P* < 0.05; high school versus college, *P* < 0.01) (Table 2(a)). The data in Table 2(b) shows that among the 460 women receiving IVF treatment, 13.7% had completed primary school, 56% high school, and 29.8% university or college. In addition, the educational levels of Chinese individuals older than 15 years are distributed as follows: 26.78% primary school, 52.82% high school, and 8.93% university or college, with the rest falling into other categories such as educational level lower than primary school or no education received. The educational levels of women are equal to or slightly lower than those of men in recent years [22]. When taking a census of the educational levels of the Chinese population into consideration, we found that the number of women seeking IVF treatment who had university or college education was approximately six times the number of women with primary school education, and there were approximately two times more women with high school educations than women with primary school educations.

Distribution of the age of infertile women in the different educational range levels is shown in Table 3. Compared with primary school and university level, women with high school education seeking treatment are younger (54% versus 35% and 38% in the range of 20–30 years old; 15% versus 21% and 24% in the range of 35–40 years old). Twelve percent of women in the range of primary school were older than 40 years.  Table 4 shows the distribution of medical indications for treatment in women with different educational levels. There is no significant difference between educational levels for major medical indications.

### 3.2. Psychological Stress among Women Undergoing IVF or ICSI

Which is the major source of stress: societal opinion, familial members' complaints, or both? Patients selected one option based on their judgments of the attitudes of family members (especially the elderly), partner relationships, and social discrimination. The results show that 68.6% of women reported that familial pressure was the major source of their psychological imbalance; only approximately ten percent of women felt pressured by public opinion, while 18.3% did not feel any stress from their families or society (Table 5).

Table 6 shows the prevalence of the symptoms of feelings of depression, anxiety, and helplessness detected by the ZDS, ZAS, and ICQ. Among all of the 460 participants, 35.2% showed normal psychological states, while 14.8% had depressive symptoms (with or without anxiety or helplessness); the proportions of patients with anxiety (with or without depression or helplessness) and helplessness (with or without depression or anxiety) were 33.3% and 39.1% respectively. There was no significant difference between the patients with anxiety versus helplessness based on educational levels. However, it was observed that there was a significant difference in the symptoms of depression between those with primary educational levels and high school or university educational levels. Eleven point six percent of women who graduated from universities or colleges and 11.1% of women who graduated from high school reported depressive symptoms, compared with 32.1% of persons with primary education levels (*P* < 0.01).

### 3.3. Correlation between Psychological Stress and the Outcome of IVF

The data on the correlation between psychological stress and the outcome of IVF is shown in Tables 7 and 8. Patients with combined emotional symptoms have been excluded to avoid skewing the data; as Table 7 shows, there was no significant difference in the mean number of oocytes retrieved after controlled ovarian hyperstimulation (COH) between women with versus without stress (14.8 ± 6.9; 13.4 ± 5.3; 14 ± 5.0; 14.6 ± 5.8, resp.). The percentage of normal fertilization and the percentage of excellent embryos were lower in the group of women with depression than in the other groups. Additionally, the final outcome of IVF (percentage of pregnancy) was significantly lower in the women with depression compared with those with normal emotional states (7% versus 44%, *P* < 0.01). Aside from depression, compared with the women with normal emotional states, the pregnancy rate in the women with anxiety was significantly lower (31% versus 44%). Data regarding the correlation between psychological stress and pregnancy shows that there was a negative correlation between scores on the ZDS and pregnancy (*r* = −0.9177; *P* < 0.01) (Table 8(a)). A similar negative correlation was found between scores on the ZAS and pregnancy (*r* = −0.9069; *P* < 0.01) (Table 8(b)). In our study, there were no differences in the outcomes of IVF between women feeling helplessness and those with normal emotional states.

## 4. Discussion

The results of this study indicate a relationship between educational level and duration of infertility prior to starting treatment, psychological stress caused by infertility, and the effects of psychological stress on the outcome of IVF.

A large global survey was conducted during World Fertility Awareness Month (2006) on approximately 17,500 individuals and revealed that knowledge about fertility and the biology of reproduction is lacking throughout the world; merely one-fourth of the participants knew how infertility is diagnosed [23]. The majority of couples who suffer from the condition live in developing countries [24]. Limited knowledge may determine when the couple begins seeking treatment (prematurely versus after a delay) [7]. The survey showed that the duration of more than half of the patients' infertilities was longer than 5 years, and the women with higher levels of education sought infertility treatment more often and earlier than those with lower educational levels. A majority of the participants with primary school education (73%) started seeking IVF after suffering from infertility for more than 5 years, while half of the women with university or college education levels sought IVF when they had endured infertility for longer than 2 years. It is assumed that limited knowledge about infertility and alternative treatments is one of the reasons, which can also be confirmed by the distribution of women's age with different levels of education. Among the women seeking treatment, there are more than half which are younger than 30 years in the range of high school and only around one third in the range of primary school. Nobody is older than 40 years with a high school educational level in this survey, while 12% of primary school women are older than 40 years. The reason that 38% of university or college women are younger than 30 and their duration of infertility is shorter than other educational level women can be that the marriage age is older in them compared to that of women with primary or high school educational levels. Distribution of major medical indications for treatment is similar among the women with different educational levels.

A survey of reproductive health-related knowledge among middle school and college students in China showed that schooling is not a major source of information for girls; most schoolgirls reported acquiring their knowledge from the Internet, magazines, and newspapers, and fewer than one-fifth reported obtaining knowledge from their parents. Lessons addressing reproductive physiology do not begin until the second level of middle school, when children are 14 or 15 years old; this age is late in most children's physical and mental development. Additionally, because of traditional Chinese views and social conventions, the lesson is not taught by teachers in most schools; students are asked to teach themselves. Parents usually avoid the subject of sex with their children for the same reason. Knowledge of reproductive health and sexual behaviour among adolescent females is important for their future fertility. Lacking such knowledge could be one of the reasons that infertility is observed to be more frequent and longer lasting in China.

The cost of infertility treatment is another reason that less-educated couples hesitate to seek treatment. Compared with more-educated women, less-educated women's incomes were lower on average. Many of them also live in the countryside (data not shown). An IVF treatment fee of more than $5,000 may pose significant financial pressure on them. As ChinaNews reports, the annual per capita income in urban areas was around $3,900 in 2011, while it was around $1,140 in the countryside [25]. In addition, medical conditions in most of China's rural areas need improvement. Faulty medical services could be another reason for infertile women not seeking IVF treatment.

Bearing progeny is regarded as part of a stable marital nexus. In China, children, particularly sons, are regarded as a continuance of an entire family and a source of income and security in old age for those with lower incomes [26]. Because of the importance of children for a family and because of limited knowledge regarding infertility, wives would often blame themselves for infertility and their husbands would feel that their dignity was lost. Moreover, because the social and economic statuses of women with lower educational levels are generally lower, most of these women have to depend on their husbands and the negative influence of infertility is more serious. Compared with more-educated women who can support themselves, less-educated women feel depression and anxiety.

This survey showed that 68.6% of the participants reported pressure from their families; in addition, 3.8% reported pressure from both family and society, which confirmed the studies from other developing countries [5]. Women are blamed for infertility by societal norms and culture. Such women are even considered unlucky—not only to their husbands but also to their entire families—because they could not help their family lines to continue [27]. In Pakistan, young newlyweds, especially women, are pressured by the elderly in the family if they are unable to conceive immediately after marriage [5]. Placement of unnecessary blame on a woman can become socially crippling for her.

Although IVF failure is still common and the procedures of treatment are uncomfortable and complicated, the probability of successful IVF/ET treatment ranges from 37.8% to 60%, and it is still considered one of the most useful options available to infertile couples [28]. Emotional instability in women, such as anxiety and depression, is considered a cause of early dropout after the first IVF cycle and is related to lower pregnancy rates. Attention to the psychological aspects present during IVF treatment is strongly advisable [29]. In a Chinese prevalence study, 17% of infertile women had major depressive disorder and 23.2% had generalized anxiety disorder [7]. The importance of the relationship between psychological stress, particularly infertility-related stress, and the outcome of IVF has been discussed in previous literature. Stressful emotional experiences were reported to be negatively associated with the success of assisted reproduction [30].

In this study, the rate of depression decreased as educational level increased. History of infertility was highly associated with depressive symptoms [14]. The less-educated women generally had longer durations of infertility, which may be one of the reasons for the higher rate of depression in women with primary school educational levels than in those with university or college education levels. Furthermore, independence and greater knowledge about fertility could be reasons behind the lower levels of depression in the more-educated women. We found that the percentages of normal fertility and excellent embryos and pregnancy rates were lower in women with depression than in other women. The negative association between baseline psychological state and outcome of IVF in this study is consistent with several previous studies [30].

## Figures and Tables

**Table 1:** Duration of infertility when women begin seeking IVF treatment.

Total	≤1 year		1-2 years		2–5 years		≥5 years	
	*n *	%	*n *	%	*n *	%	*n *	%
*N*: 460	14	3%	38	8.3%	161	35%	247	53.7%

**Table:** (a) Duration of infertility in women with different educational levels

Education level (total: 460)						
Duration of infertility	Primary school *N*: 63		High school *N*: 260		University/college *N*: 137	
	*n *	%	*n *	%	*n *	%
≤1 year	2	3%	8	3%	4	2.9%
1-2 years	6	9.5%	18	6.9%	14	10.2%
2–5 years	9	14.3%	86	33%	66	48.2%
≥5 years	46	73%	148	56.9%	53	38.7%

**Table:** (b) Ratio of the women with different educational levels seeking IVF

Patients seeking IVF or ICSI treatment (*N*: 460)					
Primary school		High school		University/college	
*n *	%	*n *	%	*n *	%
63	13.7%	260	56.5%	137	29.8%

Educational levels of Chinese individuals older than 15 years					
Total	Primary school	High school	University/ college		Others

100,000	26.78%	52.82%	8.93%		

Ratio between patients who accepted ART treatment and individuals at the same educational level					

*R *	13.7%/ 26.78% = 0.51	56.5%/ 52.82% = 1.07	29.8%/ 8.93% = 3.34		

Data regarding educational levels of Chinese individuals older than 15 years from the No. 6 Census of China's Population-the quality of population report.

**Table 3:** Distribution of the age of infertile women with different educational levels.

	Age (years)			
	20–30	30–35	35–40	>40
Primary school	22	20	13	8
*N*: 63	35%	32%	21%	12%
High school	140	80	40	0
*N*: 260	54%	31%	15%	0%
University/college	52	49	33	3
*N*: 137	38%	36%	24%	2%

**Table 4:** Distribution of medical indications for treatment in the women with different educational levels.

	Male		Female		Others
	Azoospermia	Oligospermia	Tubal obstruction	Follicle development failure	
Primary school	8	10	31	8	6
*N*: 63	11%	16%	50%	12%	11%
High school	26	44	135	36	19
*N*: 260	10%	17%	52%	14%	7%
University/college	6	12	36	9	3
*N*: 137	9%	18%	54%	13%	4%

**Table 5:** Source from which infertile women feel pressure.

Total	Family		Society		Both		None	
	*n *	%	*n *	%	*n *	%	*n *	%
*N*: 460	316	68.6%	43	9.3%	17	3.8%	84	18.3%

**Table 6:** The prevalence of symptoms of feeling depression, anxiety, and helplessness in patients with different levels of education.

Total *N*: 460	Depression ZDS ≥ 40		Anxiety ZAS ≥ 40		Helplessness ICQ ≥ 14		Normal emotion ZDS < 40 ZAS < 40 ICQ < 14	
	*n *	%	*n *	%	*n *	%	*n *	%
	68	14.8%	153	33.3%	180	39.1%	162	35.2%
	*n *	%	*n *	%	*n *	%	*n *	%
Primary school *N*: 78	25	32.1%	25	32.1%	26	33.3%	31	39.7%
High school *N*: 235	26	11.1%	79	33.6%	92	39.1%	79	33.6%
University/ college *N*: 147	17	11.6%	49	33.3%	62	42.2%	52	35.4%

ZDS: Zung Depression Scale; ZAS: Zung Anxiety Scale; ICQ: Illness Cognition Questionnaire.

**Table 7:** Correlation between psychological stress and the outcome of IVF.

*N*:	Depression 57	Anxiety 52	Helplessness 87	Normal emotion 162
Pregnancy rate	7%	31%	42%	44%
Mean number of oocytes	14.8 ± 6.9	13.4 ± 5.3	14.0 ± 5.0	14.6 ± 5.8
Normal fertilization rate	38%	61%	50%	50%
Excellent embryos rate	22%	37%	35%	38%

Normal fertilization rates: the percentage of double pronucleus zygotes formed; excellent embryos: embryo on day 3 consisted of at least 6 cells, fragment less than 10%.

**Table:** (a) Correlation between depression and pregnancy

ZDS	0–10	10–20	20–30	30–40	40–50	50–60	60–70	70–80
Pregnancy rate	50.0%	44.4%	55.0%	24.3%	10.0%	6.7%	8.3%	0%

*R* = −0.9177, *P* < 0.01.

**Table:** (b) Correlation between anxiety and pregnancy

ZAS	0–10	10–20	20–30	30–40	40–50	50–60	60–70
Pregnancy rate	54.3%	42.2%	46.7%	32.4%	31.8%	30.8%	29.4%

*R* = −0.9069, *P* < 0.01.
